# We can’t afford the candy! The influence of parenting by lying about money on mobile phone addiction and the roles of anxiety and socioeconomic status among Chinese adolescents

**DOI:** 10.3389/fpsyg.2022.1021355

**Published:** 2022-11-09

**Authors:** Hua Wei, Lijun Lu, Meiting Liu

**Affiliations:** ^1^Normal College, Qingdao University, Qingdao, China; ^2^School of Educational Science, Xinyang Normal University, Xinyang, China; ^3^Department of Social Research, University of Turku, Turku, Finland

**Keywords:** mobile phone addiction, parenting by lying about money, anxiety, socioeconomic status, adolescents

## Abstract

**Background:**

Extant research has indicated that parenting practice, such as harsh parenting, rejection, and neglect increases the risk of mobile phone addiction. However, no research to date has examined the association between parenting by lying about money and adolescent mobile phone addiction.

**Objective:**

The current study used a survey to test whether parenting by lying about money may be associated with adolescent mobile phone addiction. The mediation of anxiety and moderation of socioeconomic status were also examined.

**Materials and methods:**

We recruited 971 adolescents from five secondary schools in a city in central China. Of the participants, 448 (46.14%) were boys and 523 (53.86%) were girls (*M*_age_ = 13.63, *SD*_age_ = 1.01).

**Results:**

The results of mediation analysis indicated that parenting by lying about money positively predicted mobile phone addiction (*B* = 0.144, *p* < 0.01); parenting by lying about money positively predicted anxiety (*B* = 0.126, *p* < 0.01) and mobile phone addiction (*B* = 0.107, *p* < 0.01). Anxiety positively predicted mobile phone addiction (*B* = 0.293, *p* < 0.01). Moreover, the bias-corrected bootstrapping mediation test indicated that the process by which parenting by lying about money predicted mobile phone addiction through anxiety was significant (indirect effect = 0.037, *SE* = 0.011, 95% CI = [0.017, 0.059]).

**Conclusion:**

The current study suggests that parenting by lying about money may lead to mobile phone addiction through the mediation of anxiety. However, the effect was stronger for adolescents with higher level of socioeconomic status than their counterparts.

## Introduction

The smart phone technology is undoubtedly developing rapidly today, and as a result, mobile phones become essential in adolescent everyday life. Although mobile phones have enhanced youth lifestyle, mobile phone addiction has become a social problem that plagues many families. Studies have found that mobile phone addiction may negatively affect adolescent physical health, mental health, and academic performance (José et al., 2016; Sahu et al., 2019). Therefore, investigating the influencing factors and functioning mechanism of mobile phone addiction has important practical significance. During the pandemic, adolescents all over the world were faced with home quarantine and thus their offline social communication was largely limited. They were also required to take online courses, which would increase their frequency and time of using mobile phones. Given that the lack of offline social interaction and the high frequency of using mobile phones are closely correlated with mobile phone addiction, mobile phone addiction is becoming a major issue since the outbreak of COVID-19. Recent empirical evidence also demonstrated the severity of the situation (Alimoradi et al., 2022). Additionally, China has been launching a strict dynamic control over the affected area since the first outbreak. Therefore, we consider it significant to examine the mobile phone addiction in such setting.

Among the many influencing factors, parenting practice has attracted the attention of many researchers. They found that parenting practice, such as harsh parenting, rejection, and neglect increases the risk of mobile phone addiction (Kwaka et al., 2018; Sun et al., 2019; Zhang et al., 2021; Wei et al., 2022). The parenting practice that scholars pay attention to involve either explicitly negative emotional and intentional features (e.g., harsh parenting, rejection, and neglect) or violent behavior (e.g., harsh parenting). Moreover, academic and public concerns have underscored the negative effects of these parenting practices, so their impact on mobile phone addiction is easily to be discerned and taken seriously.

However, some parenting practices that do not involve explicitly negative emotional and intentional features or violent behavior are likely to be ignored by researchers and parents as a potential factor to mobile phone addiction, for instance, parenting by lying. Parenting by lying is defined that parents regulate their children’s emotions and behaviors through lying to them (Heyman et al., 2009; Santos et al., 2017). This study differs from previous studies of negative parenting regarding three aspects—manner, emotion, and intention (Heyman et al., 2009; Wang et al., 2016; Santos et al., 2017). First, parents’ lying does not involve physical harm upon children, which is different from previous negative parenting, for instance, harsh parenting. Parents tend to use lies as a buffer to avoid potential conflicts with children. Second, parents’ lying does not involve obvious negative emotions. When parents practice harsh parenting, rejection or neglect on children, parents usually experience strong negative emotions such as anger and frustration. And children also react in a negative way by fearing, sobbing or crying. However, parents’ lying to children usually takes place in a rather pleasant atmosphere where parents aim not to irritate the children and the children do not show obviously negative emotions at the scene. Third, parenting by lying can be neutral or even benevolent in terms of intention. Although parents’ major goal of parenting is to regulate children’s behavior, parents always have accessory goals. For example, parents practice harsh parenting partly because they want to let off anger. Differently, parents aim to maintain parent-child relationship and avoid conflicts by lying to children. They may even consider their lies to be white lies.

Not only do many parents generally believe that all lies are bad but also they consider children’s lying a violation of ethics, and sometimes even more serious than stealing and fighting (Heyman et al., 2009). However, parents often lie to their children during parenting, believing that lying is acceptable in certain situations (Heyman et al., 2009). Although parents may barely connect parenting by lying with any negative consequences to children, it is demonstrated to be positively associated with children’s internalization and externalization issues in adolescence and adulthood (Santos et al., 2017; Liu and Wei, 2020; Setoh et al., 2020; Jackson et al., 2021; Wei and Liu, 2021). Given that mobile phone addiction is a typical problem of externalization in the digital age, we assume that parenting by lying may be positively associated with mobile phone addiction.

According to previous studies, parenting by lying consists of lies to encourage healthy eating, lies about caregivers’ leaving and staying, lies to discipline children’s misbehavior and lies to regulate children’s money spending (Heyman et al., 2013; Santos et al., 2017; Setoh et al., 2020). The current research mainly focuses on parenting by lying about money and mobile phone addiction. We consider it significant to examine the current topic in the context where China’s economic strength is growing and accordingly, Chinese residents’ household purchasing power is increasing. Today, Chinese parents tend to have sufficient financial ability to buy many items for their children that parents in the past could not afford. However, parents often lie to avoid the purchases out of certain reasons, for example, not wanting their children to eat certain foods or a distaste for wasting money. They often tell their children that they do not have enough money or their families are too poor to afford the things the children want where the parents are actually economic capable of doing so (Wei and Liu, 2021; Wei et al., 2022). As a results, adolescents may doubt their parents’ love for them when their consumption needs are frustrated (Haugen, 2005), concerning that the consumerist society constantly equates spending money on children with loving them. The distrust for parents’ love may subsequently induce anxiety and eventually lead to mobile phone addiction. Moreover, we speculate that different levels of family socioeconomic status may differ in adolescents’ attribution of parental behavior. Accordingly, we assume that family socioeconomic status may moderate the association between parenting by lying about money and mobile phone addiction.

### Parenting by lying about money and mobile phone addiction

Many parents choose to buy children happiness, expressing their affection for children through purchases (Haugen, 2005). As the Chinese economy grows and its residents’ purchasing power increases, Chinese parents resort to expensive clothes and heaps of toys to show the love for their children. In addition, marketers and commercial media have spared no effort to promote materialist advice, such as “if you love her, take her to Haagen-Dazs.” Possibly, this promotion may have contributed to parents’ notion of “consumption for love.” Under the above influence, children may become aware of associating parents’ love with the gratification of material needs (Haugen, 2005). As a result, when their parents are unwilling to meet their material needs, children may wonder whether their parents genuinely love them. For example, parenting by lying about money is a behavioral strategy that parents use to avoid materially satisfying their children. Parents are inclined to claim that they happen to forget bringing any money or their family is too poor to afford the things that the children desire (Liu and Wei, 2020; Setoh et al., 2020; Wei and Liu, 2021). Although parents’ intentions are primarily benevolent as they attempt to protect children from unhealthy food or wasteful spending, children are often too immature to understand parents’ intentions behind the lies and likely to interpret the gentle rejection as the fact that their parents no longer love them. When the children reach adolescence, although they may be cognitively conscious of parents’ good intentions, they may emotionally distrust their parents due to the previous lying.

When children do not feel enough emotional attachment from their parents, they may seek it elsewhere. Nowadays, adolescents often turn to the Internet for the feelings of being loved and respected. Once the needs are met on the Internet, those adolescents who have experienced parenting by lying about money are likely to think that the real world is not as good as the online world, which is a maladaptive cognition (Davis, 2001). According to the cognitive-behavioral model of pathological Internet use, maladaptive cognition induces constant seek for gratification on the Internet, which leads adolescents to excessive dependence on the Internet (Davis, 2001). Given that the current Chinese adolescents access the Internet mainly via mobile phones, we assume that adolescents who have experienced parenting by lying about money are likely to develop mobile phone addiction due to over-reliance on mobile phones. Moreover, previous empirical evidence demonstrated that parenting by lying about money is positively associated with parent-child attachment (Liu and Wei, 2020), and that parent-child attachment is negatively associated with mobile phone addiction (Xie et al., 2019). Therefore, parenting by lying about money may increase the risk of mobile phone addiction in adolescents through parent-child attachment. Thereby, we propose H1:

Parenting by lying about money is positively associated with mobile phone addiction.

### The mediation of anxiety

According to cognitive-motivational-relational theory of emotions (Lazarus, 1993), we consider anxiety to be a potential mediator on the association between parenting by lying about money and mobile phone addiction. We first assume that parenting by lying about money may be associated with anxiety. The cognitive-motivational-relational theory of emotions argues that people may feel anxious as a response to the uncertain threats that they encounter in certain situations (Lazarus, 1993). When adolescents experience parenting by lying about money, they may be threatened with a loss of both parents’ love and parents’ honesty. Such threats endangers adolescents outlook on life and the world, which may generate anxious symptoms. Specifically, as children increasingly expect parents to satisfy their material needs (Haugen, 2005), they may feel a loss of their parents’ love when their purchasing requests are frustrated by lies. In addition, as parents always preach honesty in children’s moral education and parenting by lying about money reveals an inconsistency between parents’ words and behavior, children may identify hypocrisy and their belief about their parents’ honesty may be threatened. Moreover, children may develop distrust about life and the world through interactions with their dishonest parents (Schweitzer et al., 2006; Cargill and Curtis, 2017), which may generate anxiety about coping with difficulties in life. Adolescents are not yet able to be completely independent, so they need the support of their parents in many aspects, especially when encountering pressure and emotional turmoil from family, school and society. Without the perception of having reliable parents at their back, a healthy life prospect of adolescence will be greatly threatened.

Secondly, anxiety may be associated with mobile phone addiction. Anxiety is an unpleasant emotion, a negative emotion that people tend to eliminate. Mobile phones, on the one hand, are functional and portable, therefore, serving as a unique tool of relieving anxiety (Melumad and Pham, 2020). On the other hand, users have access to various exciting activities on the Internet (e.g., online games and live streaming) through mobile phones, which can help to distract their attention from daily problems, thereby alleviating anxiety. However, if individuals habitually alleviate anxiety through mobile phones, they will develop maladaptive cognition, holding that the online world is happier and better than the real world. In turn, this cognition bias may increase the behavioral frequency of alleviating anxiety via mobile phones. According to the cognitive behavioral model of pathological Internet use (Davis, 2001), through the above-mentioned cyclical process, individuals will become increasingly obsessed with mobile phones, and eventually develop into mobile phone addiction. Consistent with the above reasoning, empirical research also demonstrates that the more anxious individuals are, the higher the degree of mobile phone addiction (Kim and Koh, 2018; Jon et al., 2020). Thereby, we propose H2:

Anxiety mediates the association between parenting by lying about money and mobile phone addiction.

### The moderation of family socioeconomic status

According to the attribution theory, we consider that family socioeconomic status may moderate the association between parenting by lying about money and mobile phone addiction. The attribution theory holds that different attributions for the same event will lead to different consequences (Weiner, 1995). How children react to parenting by lying about money may largely relate to children’s attribution of parents’ behavior. Children’s perception of family socioeconomic status reflect their evaluation of the purchasing power of the family, which subsequently affect adolescents’ attribution of parenting by lying about money. Specifically, adolescents from less affluent families may not blame parents for lying, as they understand that their parents have no choice but to lie and focus family expenditure on necessities with limited financial capability. In contrast, adolescents from more affluent families have little clues to justify parents’ lying, so they tend to make negative attribution of parents’ lying. They may think that their families are affluent enough to afford the purchases so that parents’ lying about money can be none other than a manifestation of not loving them. Additionally, research have found that lies lead to negative outcomes when the tellers’ intentions are believed to be benign (Fu et al., 2015; Levine and Schweitzer, 2015). In conclusion, compared with their more affluent counterparts, adolescents from less affluent families may not develop maladaptive cognition when they have experienced parenting by lying about money, and therefore less likely to develop mobile phone addiction.

**FIGURE 1:**
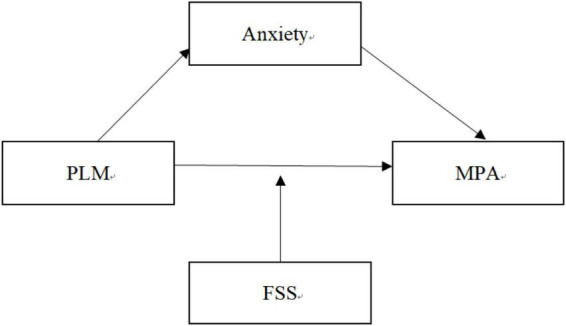
The hypothetical model. PLM, parenting by lying about money; MPA, mobile phone addiction; FSS, family socioeconomic status.

In conclusion, this study aims at examining the association between parenting by lying about money and mobile phone addiction and testing the mediation of anxiety and moderation of family socioeconomic status (Figure 1).

## Materials and methods

### Participants

The software G*Power was adopted to determine the sample size required for a small effect (0.1). The output shows that 891 participants per condition were required for the study to be powered at 85% when α ≤ 0.05. We recruited 971 adolescents from five secondary schools in a city in central China. We adopted convenience sampling in the current study. Of the participants, 448 (46.14%) were boys and 523 (53.86%) were girls (*M*_age_ = 13.63, *SD*_age_ = 1.01). Table 1 illustrates the demographic characteristics of the participants.

**TABLE 1:** Demographic characteristics of the participants.

	No.	Percentage
**Gender**		
Male	448	46.14%
Female	523	53.86%
**Sibling status**		
Only child	76	7.83%
Having one or more siblings	895	92.17%
**Place of origin**		
Urban	265	27.29%
Rural	706	62.71%

### Procedure

Before the formal investigation, we received permission to conduct this survey from the Ethics Committee for Scientific Research of our institution, and we sought consent from the participants concerning the use of their responses in our research. Before collecting data, we took steps to ensure that the participants fully comprehended the entire survey procedure, such as describing the instructions, explaining obscure items to some participants, and declaring the voluntary nature and confidentiality of participation. Furthermore, we asked the participants to check the completeness of their questionnaire responses upon finishing. The participants then completed a questionnaire that gauged their demographic characteristics, parenting by lying about money, anxiety and mobile phone addiction.

### Measures

#### Parenting by lying about money

We assessed parenting by lying about money with a Chinese version of a 4-item from the 16-item parenting by lying scale (Liu and Wei, 2020; Setoh et al., 2020; Wei and Liu, 2021). For each item, the participants were asked whether they were told such a lie by their parents, with the response options of “yes,” “no,” or “I don’t remember.” In accordance with the procedures used in previous studies, “yes” responses were totaled to indicate the degree to which the participant experienced parenting by lying, with higher scores suggesting higher exposure to parenting by lying about money in childhood. In this study, the Cronbach’s α was 0.78.

#### Anxiety

We measured anxiety with the 7-item anxiety subscale from the 21-item Depression Anxiety Stress Scale (DASS), a short version of the 42-item DASS (Lovibond and Lovibond, 1995). The anxiety subscale was rated on a Likert-type from “0 = not agree at all” to “3 = totally agree.” The present sample’s Cronbach’s α was 0.88 for anxiety.

#### Mobile phone addiction

Mobile phone addiction was measured by the Mobile phone addiction Scale (Hong et al., 2012). This scale consists of 11-items rated on a five-point scale (1 = never, 5 = always). Cronbach’s α coefficient for this scale in our study was 0.90.

#### Family socioeconomic status

We used the Family Affluence Scale (Currie et al., 2008; Liu et al., 2012; Liu and Wei, 2020) to assess the family Socioeconomic Status (SES). The scale included answers for the following four items: ownership of a family car, van, or truck (1 = none, 2 = one, 3 = two, etc.), own bedroom (no = 1, yes = 2), travel times during the past 12 months or more (1 = none, 2 = one, 3 = two, etc.), and family computer(s) (1 = none, 2 = one, 3 = two, etc.). We calculated the total scores, ranging from 4 (low affluence) to 13 (high affluence), by adding the scores of all four items, with higher total scores indicating higher SES. In this study, the Cronbach’s alpha was 0.50 which were in line with previous studies in Chinese children and adolescents (Chen et al., 2016; Liu and Wei, 2020).

### Statistical analyses

First, before the test, we employed several approaches to reduce common method bias, including participant anonymity, rearrangement, and reverse expression of items. In addition, we employed Harman’s single factor test to determine whether common method bias exists in this study. The results showed a multiple-factor structure, and that the largest loading factor only accounted for 26.41% of the total variance, far less than the 40.00% threshold, suggesting no significant common method bias in this study (Podsakoff et al., 2003).

Second, descriptive analysis was performed to examine the participants’ characteristics regarding the studied variables, and Pearson correlation analysis was performed to examine the correlations between variables.

Third, the present study used PROCESS version 3 (Hayes, 2013) to test the mediating and moderated model. Given that PROCESS Marco does not provide standardized regression coefficients, we calculated z-scores before data analyses. We generated 5,000 bootstrapped samples to approximate the confidence interval (CI) of the indirect effect based on the original sample (*n* = 971). A 95% bias-corrected accelerated CI without zero indicates statistical significance. In addition, the age and gender of the participants were controlled in the analyses.

## Results

### Preliminary analyses

Table 2 presents the Pearson correlations, means, and standard deviations of all variables. As Table 2 indicates, Parenting by lying about money was positively correlated with anxiety and mobile phone addiction. Anxiety positively correlated with mobile phone addiction.

**TABLE 2:** Means, standard deviations, and correlations for the main variables (*N* = 971).

Variables	*M*	*SD*	1	2	3	4
1. Parenting by lying about money	0.377	0.374	−			
2. Anxiety	1.945	0.756	0.123**	−		
3. Mobile phone addiction	3.224	1.056	0.131**	0.289**	–	
4. Family socioeconomic status	7.031	1.804	0.021	0.056	−0.065*	–

*p < 0.05, **p < 0.01.

### Mediation analyses

We used Model 4 of PROCESS (Hayes, 2013) to examine the possible association between parenting by lying about money and mobile phone addiction as well as the possible mediating effect of anxiety. The results of the mediation analysis are presented in Table 3. After controlling for age, gender, we first found that parenting by lying about money positively predicted mobile phone addiction, *B* = 0.144, *p* < 0.01 (Eq. 1). Second, parenting by lying about money positively predicted anxiety, *B* = 0.126, *p* < 0.01 (Eq. 2). Third, parenting by lying about money positively predicted mobile phone addiction *B* = 0.107, *p* < 0.01, anxiety positively predicted mobile phone addiction, *B* = 0.293, *p* < 0.01 (Eq. 3). Finally, the bias-corrected bootstrapping mediation test indicated that the process by which parenting by lying about money predicted mobile phone addiction through anxiety was significant, indirect effect = 0.037, SE = 0.011, 95% CI = [0.017, 0.059]. The results of the mediation analysis support H1 and H2.

**TABLE 3:** The mediation model.

Predictor	Equation 1 (criterion = MPA)		Equation 2 (criterion = Anxiety)		Equation 3 (criterion = MPA)	
	*B*	*t*	*B*	*t*	*B*	*t*
PLM	0.144	4.377**	0.126	4.069**	0.107	3.356**
Anxiety					0.293	8.910**
Gender	–0.136	−2.294*	0.114	2.032*	–0.169	2.961**
Age	0.085	2.856**	0.109	3.904**	0.053	1.836
*R* ^2^	0.030		0.035		0.032	
*F*	10.088**		11.672***		28.028**	

*p < 0.05, **p < 0.01. PLM, parenting by lying about money; MPA, mobile phone addiction.

### Moderation analyses

**FIGURE 2:**
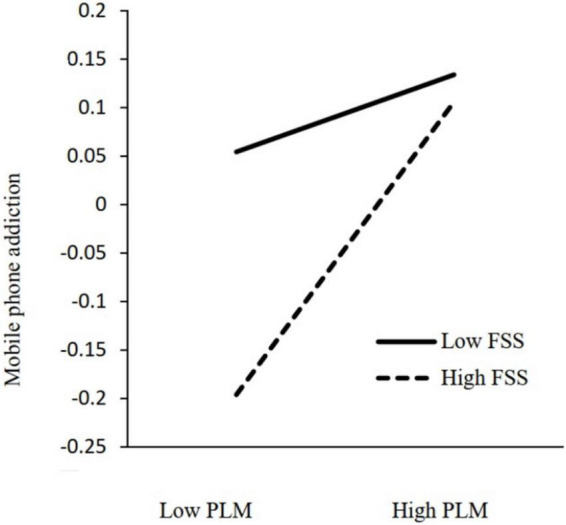
The interaction of parenting by lying about money and family socioeconomic status on mobile phone addiction. PLM, parenting by lying about money; FSS, family socioeconomic status.

We employed Model 5 of PROCESS (Hayes, 2013) to investigate whether family socioeconomic status moderated the association between parenting by lying about money and mobile phone addiction. The results of the moderation analysis can be found in Table 4 and Figure 2. The regression model indicated that the interaction between parenting about money by lying and family socioeconomic status was associated with depression (*B* = 0.066, *p* < 0.05).

**TABLE 4:** The moderation model.

Predictors	Equation (criterion = MPA)	
	*B*	*t*
PLM	0.105	2.403*
Anxiety	0.298	9.092**
FSS	–0.077	−2.515**
Gender	–0.171	–3.014
Age	0.043	1.495
PLM × FSS	0.066	2.037*
*R* ^2^	0.113	
*F*	20.541**	

*p < 0.05, **p < 0.01. PLM, parenting by lying about money; MPA, mobile phone addiction; FSS, family socioeconomic status.

**FIGURE 3:**
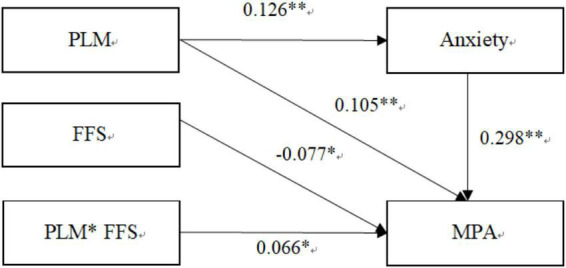
The Integrated Model. PLM, Parenting by lying about money; MPA, Mobile phone addiction; FSS, Family socioeconomic status. Path values are the path coefficients, *p < 0.05, ^**^p < 0.01.

Simple slope tests revealed that for adolescents with lower levels of family socioeconomic status, parenting by lying was positively associated with depression (*b* simple = 0.168, 95% CI [0.082, 0.254]), *p* < 0.01), while for adolescents with higher levels of family socioeconomic status, this association was not significant (*b* simple = 0.044, 95% CI [−0.043, 0.131], *p* > 0.05). Figure 3 illustrates the interaction plot.

## Discussion

### Parenting by lying and mobile phone addiction

The result indicated that parenting by lying was positively associated with mobile phone addiction, which demonstrated H1. This result is consistent with previous results that negative parenting practice, including harsh parenting, rejection and neglect, is positively associated with mobile phone addiction (Kwaka et al., 2018; Sun et al., 2019; Zhang et al., 2021; Wei et al., 2022). Therefore, the current study expanded our understanding of negative parenting practice.

Different from explicitly negative parenting practice, parenting by lying has its emotional and behavioral characteristics that result in less attention drawn from parents. First, explicitly negative parenting practices, for example, harsh parenting, rejection and neglect, usually involves intensely negative emotions, such as anger and disappointment. Parents are likely to be aware of and reflect on the harm of these practices *ex post*. In contrast, parenting by lying is not necessarily accompanied by negative emotions and is usually implemented when parents are emotionally stable. Therefore, they tend to regard the practice as reasonable strategy to regulate children’s emotions and behaviors and pay little attention to the potential negative consequences (Heyman et al., 2009). Second, practice as harsh parenting is usually engaged in physical violence to which parents and the public have been alert, so it is easier for them to realize that harsh parenting is problematic and be more conscious of it in the future parenting practice. However, parenting by lying is often carried out by parents while coaxing their children. As they often use a gentle tone to tell lies that they consider to be “white,” it is difficult for them to discern the problems.

Although few parents are aware of the dangers of parenting by lying, the results of this study found a significant association between parenting by lying about money and adolescent mobile phone addiction. Along with previous studies on the negative effects of parenting by lying (Liu and Wei, 2020; Setoh et al., 2020; Jackson et al., 2021; Wei and Liu, 2021), we consider it meaningful to draw more attention to the negative outcomes of parenting by lying. Furthermore, with the prevalence of smart phones, mobile phone addiction is increasingly developing into a major problem in both family and school education. The results of this study remind parents that if they want to reduce the risk of their children’s mobile phone addiction, they can no longer ignore the seemingly harmless parenting by lying.

### The mediation of anxiety

The result demonstrated H2 and indicated that parenting by lying about money was positively associated with mobile phone addiction through the mediating role of anxiety. This result adds to an expanding body of literature suggesting that problematic parenting practice is associated with mobile phone addiction through the mediating role of negative emotions. It also expands prior literature by demonstrating that adolescent emotional and behavioral adaptive problems can be associated with parenting practice that appears to have no intensely and instantly negative emotions and violence.

The result also expands our understanding on the functioning mechanism regarding how parenting by lying is associated with externalization problems. Previous studies explains this association through the social learning theory, arguing that parenting by lying may increase children’s frequency of lying to parents, which consequently leads to externalization problems. In this respect, this study explored a different perspective by introducing an emotion-related factor to this association. Compared to the mediating factor that adopted in the previous study (Setoh et al., 2020), the mediation of anxiety underlines its significance to psychotherapy. Given that practitioners have used emotion regulation therapy to intervene addiction problem (Azizi et al., 2010; Zargar et al., 2019), the current result suggests that this could also be employed in helping adolescents who suffer from mobile phone addiction.

### The moderation of family socioeconomic status

The result demonstrated H3 and found that family socioeconomic status moderate the association between parenting by lying about money and mobile phone addiction. Specifically, adolescents with more family socioeconomic status are likely to develop mobile phone addiction when having experienced parenting by lying about money, whereas no significant association holds for adolescents with less family socioeconomic status. This result is interesting and counters to the protective effect of family socioeconomic status revealed in previous studies. Many previous studies have found that well-off family environment is of both direct and indirect advantages for individuals. For example, Individuals from higher socioeconomic background are less likely to develop mobile phone addiction than those from lower background (Lin and Liu, 2020). Additionally, some researchers have found that socioeconomic status buffers the association between the frequency of using social networks and Internet addiction (Jin et al., 2017). In Jin et al.’s (2017) study, individuals with lower socioeconomic status have higher frequency of using social networks and therefore more likely to develop Internet addiction, whereas no significant association holds for individuals with higher socioeconomic status. Our current study, however, has reverse results, suggesting a new way of looking at the effect of socioeconomic status. It is likely that family socioeconomic status may function differently to children in view of different parenting practices. Moreover, our result recommends that families with higher socioeconomic status be more cautious of or avoid parenting by lying about money. Instead, parents can guide children’s consumer behavior by parental communication and increasing children’s self regulation in general.

Finally, the current study enriched the study of the moderators on the association between parenting by lying and adolescent adaptive problems. Previous research examined a subjective factor, parent-child attachment, that moderates the association between parenting by lying and depression (Wei and Liu, 2021). The current study examined an objective factor as the moderator on the association, providing some insights into future research.

### Limitations and implications

The first limitation was that we merely studied adolescents from mainland China, and therefore, it is not possible to generalize the results to the global population. Given that the level of adolescents’ family socioeconomic status differs in the countries with different economic development levels and the attitude toward money varies in different cultures, the current results may not be drawn when conducting the same study in different countries and cultures. Therefore, future studies can consider cross-cultural comparisons. The second limitation was that we merely collected homologous data, which may create shared variance among the studied variables and overstated the effects of hypothesized associations. In the future research, multiple sources of participants, for example parents and peers, can be recruited to report on the same variables. The third limitation was that the current study adopted a cross-sectional study design so that no causal relationships can be demonstrated. Longitudinal studies can be conducted to illustrate the directions between parenting by lying about money, anxiety and mobile phone addiction.

Practical implications could also be drawn from this study. Despite the prevalence of parenting by lying about money in Chinese society, the concept is unfamiliar to the public and people are rarely conscious of its negative effect. Thereby, the government can advertise the phenomenon via social media or traditional media to raise parents’ awareness and reduce the negative parenting behavior. Moreover, governments and schools can give guidance to adolescents to make appropriate attribution for parents’ behavior. Parents who lie to their children about money sometimes intend for protecting children’s health (eat less candy) or parents are reluctant to tell the children the disadvantaged situation of their family economic status. If children are aware of these intentions, the negative effect of parenting by lying about money may be dampened. In addition, given that anxiety and mobile phone addiction can be successfully intervened through psychological consulting or group therapy, governments and schools can organize these activities to help adolescents nurture their mental health. For those areas where psychological help resource is limited, governments and schools can organize online activities to align with their needs.

## Data availability statement

The raw data supporting the conclusions of this article will be made available by the authors, without undue reservation.

## Ethics statement

The studies involving human participants were reviewed and approved by the Research Ethics Committee of School of Educational Science, Xinyang Normal University. Written informed consent to participate in this study was provided by the participants or their legal guardian/next of kin.

## Author contributions

HW and ML designed the work and responsible for the overall development of this study, including the planning of sample collection, data analysis, writing, and polishing of the manuscript. HW, LL, and ML were in charge of data collection, analysis of this study and main revision for this manuscript, responsible for revising the manuscript, and made a great contribution to the final acceptance of the manuscript. ML provided the manuscript fee. All authors contributed to the article and approved the submitted version.
